# Visualizing the Human Subcortex Using Ultra-high Field Magnetic Resonance Imaging

**DOI:** 10.1007/s10548-018-0638-7

**Published:** 2018-03-02

**Authors:** M. C. Keuken, B. R. Isaacs, R. Trampel, W. van der Zwaag, B. U. Forstmann

**Affiliations:** 1 Integrative Model-Based Cognitive Neuroscience Research Unit, University of Amsterdam, Postbus 15926, 1001NK Amsterdam, The Netherlands; 2 Cognitive Psychology Unit, Institute of Psychology and Leiden Institute for Brain and Cognition, Leiden University, Leiden, The Netherlands; 3 Maastricht University Medical Center, Maastricht, The Netherlands; 4 Max Planck Institute for Human Cognitive and Brain Sciences, Leipzig, Germany; 5 Spinoza Center for Neuroimaging, Amsterdam, The Netherlands; 6 Netherlands Institute for Neuroscience, An Institute of the Royal Netherlands Academy of Arts and Sciences, Amsterdam, The Netherlands

**Keywords:** Subcortex, Ultra-high field, Magnetic resonance imaging, Review

## Abstract

With the recent increased availability of ultra-high field (UHF) magnetic resonance imaging (MRI), substantial progress has been made in visualizing the human brain, which can now be done in extraordinary detail. This review provides an extensive overview of the use of UHF MRI in visualizing the human subcortex for both healthy and patient populations. The high inter-subject variability in size and location of subcortical structures limits the usability of atlases in the midbrain. Fortunately, the combined results of this review indicate that a large number of subcortical areas can be visualized in individual space using UHF MRI. Current limitations and potential solutions of UHF MRI for visualizing the subcortex are also discussed.

## Introduction

**Fig. 1:**
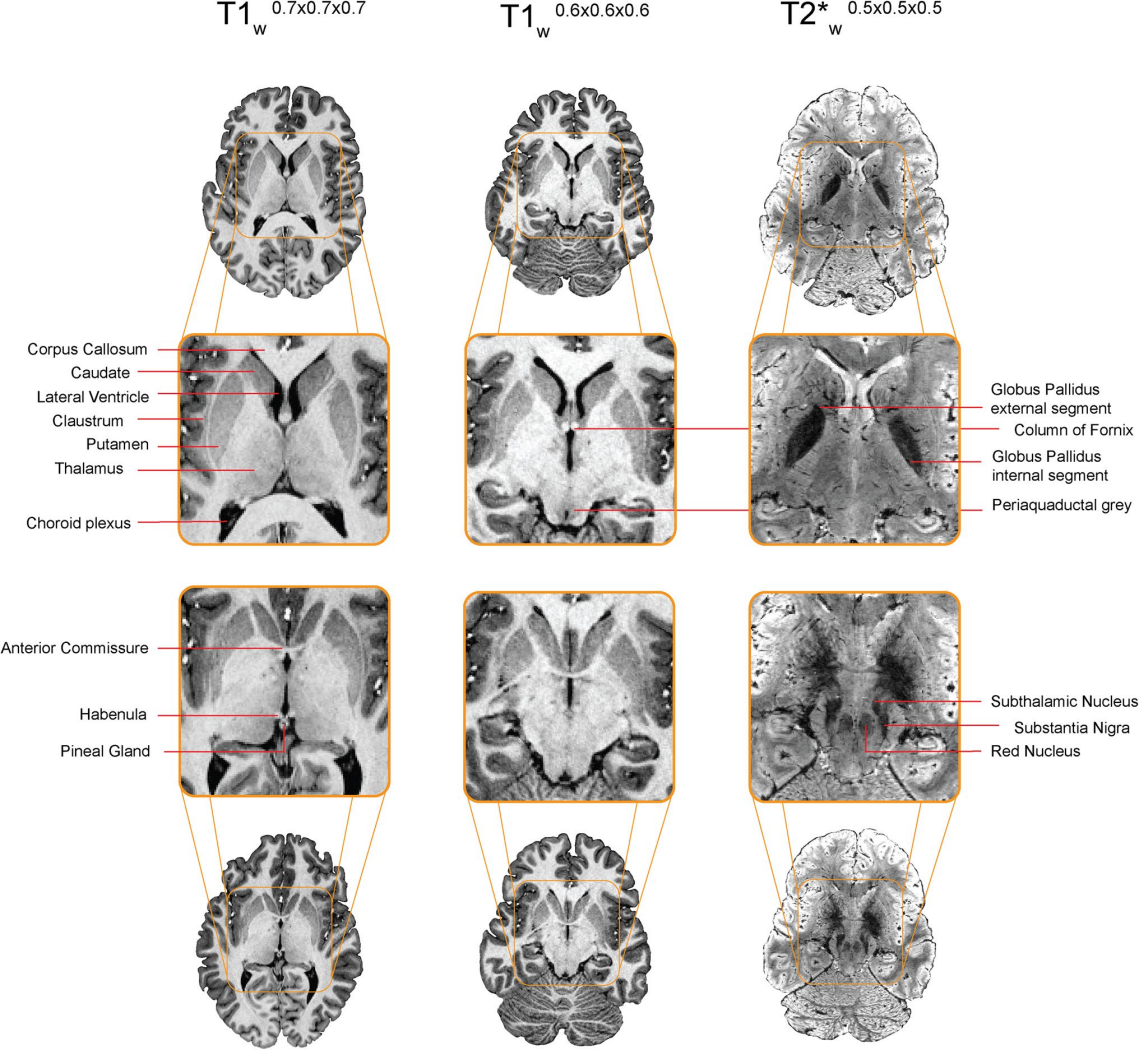
A visualization of a number of subcortical nuclei. Note that a number of nuclei, such as the STN, barely show any contrast on the T_1_-weighted scans but are clearly visible on the T_2_*-weighted scans. Image is adapted from (Forstmann et al. 2014)

In the last 25 years, the number of ultra-high field (UHF) (7.0 T and higher) magnetic resonance imaging (MRI) scanner sites has steadily increased globally (> 70 UHF MRI scanners worldwide at the time of writing). Previous reviews have highlighted the benefits of UHF MRI in the clinical domain (Beisteiner et al. 2011; van der Kolk et al. 2013; Kraff et al. 2014; Benjamin et al. 2015), in functional (f)MRI (Barth and Poser 2011; Francis and Panchuelo 2014), and in the visualization of specific subcortical structures such as the basal ganglia (BG) (Plantinga et al. 2014). For the subcortex as a whole, ultra-high field imaging is especially important, because of the possibility of identification and parcellatation of subcortical structures per individual. The use of atlases is well-spread for the larger cortical and subcortical regions, but atlases only exist for a relatively small number of the subcortical structures (Alkemade et al. 2013). In addition the size and location of subcortical regions vary substantially between individuals (Keuken et al. 2014; Tona et al. 2017), necessitating visualization of these areas in individual space. The subcortex is approximately five times smaller than the neocortex but consists of a large number of unique subcortical structures [approximately 455 structures (Dunbar 1992; Federative Committee on Anatomical Terminology 1998; Alkemade et al. 2013; Forstmann et al. 2017a)]. See Fig. 1 for a number of subcortical structures.

As noted by Johansen-Berg, recent empirical studies on human cognition seem to neglect this part of the brain (Johansen-Berg 2013). To understand how cognitive functions are implemented in the brain, it is, however, vital to study the entire network of structures that might be functionally involved. The so called cortical-basal ganglia-thalamic loops exemplify how studying both cortical and subcortical areas is essential for fully understanding cognitive function (Alexander and Crutcher 1990; Alexander et al. 1990; Haber and Calzavara 2009; Ding and Gold 2013). These structural loops have a general topographic organization, whereby distinct cortical areas project to both the striatum (STR) and subthalamic nucleus (STN). The STR and STN are strongly connected to other BG nuclei, which via thalamic sub-nuclei project back to the cortex. It is thought that as a result of these distinct structural connections, the cortical-BG-thalamic loops are involved in motor, limbic, and cognitive functions (Alexander et al. 1990; Middleton and Strick 2000a; Haber and Calzavara 2009). For instance, within the thalamus the motor loop projects from the cortical motor areas to the ventral lateral nucleus pars oralis, whereas the cognitive loops, involving cortical areas such as the DLPFC, are thought to involve the directly adjacent ventral anterior nucleus pars parvocellularis (Middleton and Strick 2000b). To be able to study these functional domains it is therefore crucial to separate the distinct areas in the subcortex just as it is essential to identify the structural and functional distinct cortical areas (Turner 2013; Turner and Geyer 2014; Forstmann et al. 2017a).

With the increase of field strength, substantial progress has been made in visualizing the human brain in extraordinary detail (Robitaille and Berliner 2007; Duyn 2010; van der Zwaag et al. 2015; Cho 2016; Setsompop et al. 2016; Marrakchi-Kacem et al. 2016; Budinger et al. 2016; Turner and De Haan 2017; Dumoulin et al. 2017; Marques et al. 2017; Giuliano et al. 2017; Sclocco et al. 2017; Kemper et al. 2017; Gallichan 2017). Using UHF MRI, it has become possible to visualize intracortical anatomical structures, such as the bands of Baillarger, in vivo where before they could only be identified using post mortem myelin stains (Turner 2011; Fracasso et al. 2016).

Generally however, imaging the human subcortex with MRI has been particularly challenging for a number of reasons (Forstmann et al. 2017a). The subcortex consists of a large number of small, directly adjunct structures of which a large number have anatomical properties that makes them very hard to distinguish with standard anatomical T_1_-weighted MRI and require tailored MRI contrasts (Tourdias et al. 2014; Visser et al. 2016a; Priovoulos et al. 2017). Other general MRI factors that hinder the visualization of the subcortex include the substantially lower absolute SNR in the middle of the brain than in the cortex due to the increased distance from the elements of the modern head coils (Wiggins et al. 2009; de Hollander et al. 2017). In addition, g-factor penalties associated with parallel imaging, are larger in the middle of the brain (Larkman 2007; Vaughan and Griffiths 2012; Pohmann et al. 2015).

The visualization of small subcortical structures benefits from UHF for a number of reasons. The first is the linear increase of signal-to-noise ratio (SNR) with field strength (McRobbie et al. 2006; Robitaille and Berliner 2007; Duyn 2012; van der Zwaag et al. 2015; Pohmann et al. 2015). This increased SNR can be used to improve the spatial resolution and visualize fine grained details due to reduced partial volume effects (PVE) (Lüsebrink et al. 2013; Federau and Gallichan 2016). Further, UHF MRI can provide increased T_1_-contrast between grey and white matter (van der Zwaag et al. 2015). Similarly, T_2_* differences tend to be larger at 7T than at lower fields, leading to larger contrasts which has been used for the identification of anatomical borders between the substantia nigra (SN) and STN which were previously challenging to visualize (Dula et al. 2010; Abosch et al. 2010; Cho et al. 2011b). Finally, the g-factor penalties in the middle of the brain are lower on 7T than on 3T, which means that higher acceleration factors can be achieved on 7T with a smaller SNR loss than on 3T (Wen et al. 2015). These advantages of UHF MRI make it a powerful tool for visualizing small nuclei in vivo.

Using UHF MRI several of the thalamic subnuclei can now be visualized in individual space without the need to refer to standardized atlases (Tourdias et al. 2014; Saranathan et al. 2014; Kanowski et al. 2014). However, a large and growing number of subcortical structures can be visualized using UHF MRI, many of which have been demonstrated in a single publication. This paper provides and overview of the 169 subcortical structures which have so far been visualized in the human brain using UHF MRI and the methods used to achieve this. The review will focus on the type of MRI sequence, participant demographics and methods used to parcellate the structure of interest.

## Materials and Methods

### Search Strategy

A comprehensive literature search was conducted using the Entrez search tools implemented in the Biopython’s *Bio.Entrez* module (Cock et al. 2009). This is a python application programming interface (API) tool that queries the PubMed database (http://www.pubmed.org). The query date was the 1st of December 2017 and used the following inclusion criteria: publication date was before the 1st of December 2017, focused on humans, used an MRI scanner with a static B_0_ field strength ≥ 7.0 T, and report the visualization of a subcortical (either in the cerebrum, cerebellum or brainstem) nucleus or region. The search terms that were used were for example “ultra-high field magnetic resonance imaging”, “7 T structural MRI”, “7T neuroimaging”, and “7.0 T magnetic resonance imaging”. All search terms were used with the different common B_0_ field strengths for UHF MRI (7.0, 8.0, 9.4, 10.5, and 11.7).

### Inclusion Procedure

All 5818 resulting abstracts were read by two raters (MCK & BRI) and based on the inclusion criteria detailed above, a decision was made to read the full-text paper or not. The abstracts that both raters did not agree on were checked again. The potential 388 full-text papers were read by a single rater (MCK) and were separated into reviews and empirical papers. The 299 empirical papers were checked for all inclusion criteria and if there was a match, the paper was included in the final list. The 58 review papers were cross referenced, which entailed that the 5252 abstracts of all cited papers were read and checked for additional potential full-text papers.

**Fig. 2:**
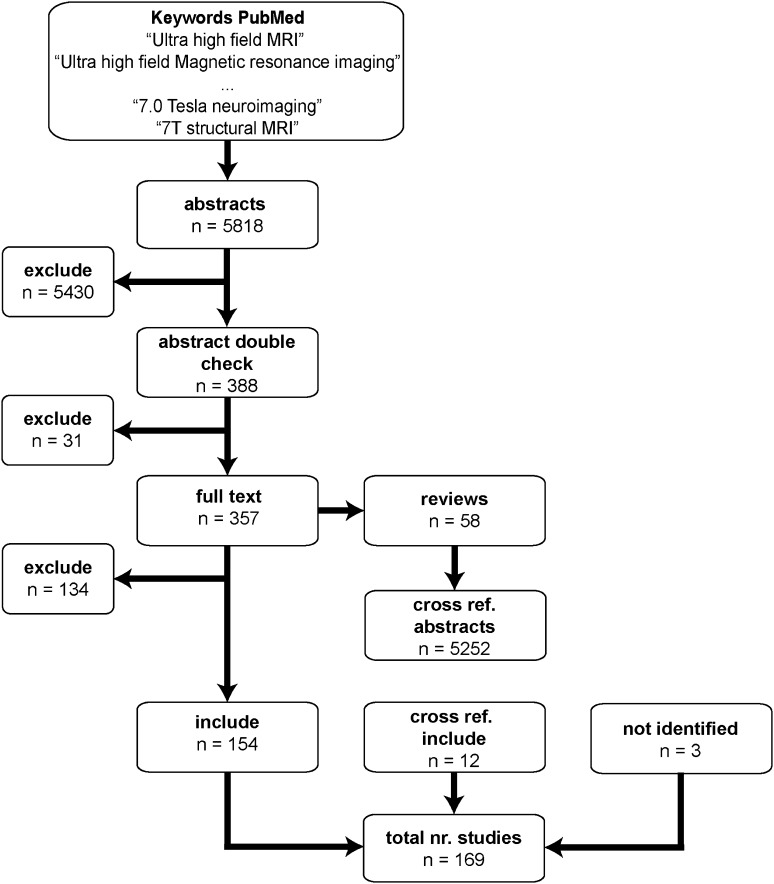
Search strategy. Using the Entrez search tools implemented in the Biopython’s *Bio.Entrez* module the PubMed database was queried for a number of search terms. This resulted in a number of abstracts that were read and double-checked by two independent readers. The resulting full texts were then downloaded and separated in empirical studies and reviews. The empirical papers were read to check if they matched the inclusion criteria, resulting in the inclusion of 131 papers. The reviews were cross referenced and resulting abstracts were read by one rater. The resulting full text empirical papers were read, and an additional 9 papers were added. Finally, the 140 papers from the PubMed search were compared to the publications by the authors of this review. This resulted in 2 papers that were not identified by our search strategy

Finally, to test whether the employed search strategy resulted in a comprehensive set of papers, the included papers were compared to the publications of the authors of this review. The included papers were compared to the list of publications which were a priori known to fit the inclusion criteria. This comparison indicated that two out of the 27 papers by our own group were not found via the PubMed search, implying that approximately 7% of the empirical papers that would fit the inclusion criteria were not identified. The literature search resulted in the inclusions of 169 papers (see Fig. 2 for an overview of the article selection procedure).

The information extracted from the papers was as follows: which subcortical structures were visualized or parcellated, whether the measurements were from in vivo or post mortem samples, whether the population consisted of healthy or clinical subjects, which MRI contrast was used to visualize the subcortical structures, and the accompanying MRI parameters.

#### Identification Versus Parcellation

The subcortical structure(s) in each paper was classified as being either ‘identified’ or ‘parcellated’. Identification was defined as the placement of abbreviations, arrows or other visual markers that corresponded to an anatomical label in an image of a structural MRI scan. Parcellation was defined as the manual, automatic, or semi-automatic delineation of the entire or partial structure. Manual parcellation is defined as the process where an expert delineates and labels the borders of a region of interest (ROI) manually [e.g., (Lenglet et al. 2012; Kwon et al. 2012)]. Automatic parcellation is defined as the process where the ROI is parcellated using a software package without any manual editing [e.g., (Zhang et al. 2001; Visser et al. 2016a)]. Semi-automatic parcellation is defined as automatic parcellation whereby the resulting parcellation is manually edited if needed [e.g. (Mestres-Missé et al. 2014)].

The parcellation method had to employ the actual contrast of the nuclei and the surrounding tissue. Single atlas label propagations, where an individual anatomical MRI scan is registered to a pre-labeled standard structural template, were excluded. The reason for this exclusion is that label propagation is a registration problem between the template and the entire individual anatomical MRI volume and is unable to capture large anatomical variation (Doan et al. 2010; Cabezas et al. 2011).

#### MRI Sequence Classes

The MRI contrasts which were used to visualize the structures of interest were grouped according to the main classes of contrasts: T_1_, T_2_, T_2_*, functional (regardless of underlying mechanism—T_2_* BOLD, T_2_ BOLD, T_1_ VASO, fQSM, etc), diffusion weighted imaging (DWI), susceptibility weighted imaging (SWI), including phase imaging and quantitative susceptibility mapping (QSM), magnetization transfer (MT), proton density (PD), multiple, and other. The multiple MRI sequence category entails those studies that visualized the structure of interest in a number of MRI sequences. Inclusion in the ‘other’ category was either a single MRI sequence that was not specific to a given contrast mechanism (e.g., both PD and T_2_ weighted) or did not fit the above classification scheme (e.g., magnetic resonance spectroscopy).

It is beyond the scope of this review to go into a detailed description of the separate contrast mechanisms and we refer to the following literature (McRobbie et al. 2006; Robitaille and Berliner 2007). Very briefly, a T_1_ contrast is based on the recovery time of the longitudinal component of the magnetization following the application of a radio frequency excitation pulse, while T_2_ refers to the decay of the transverse magnetization component as a result of proton interactions (McRobbie et al. 2006). The T_2_* contrast is based on the decay of the transverse magnetization component as a result of proton interactions and the magnetic field inhomogeneity (McRobbie et al. 2006; Chavhan et al. 2009). The DWI contrast is based on the dephasing of the protons due to the diffusion of water molecules (Jones et al. 2013; Chilla et al. 2015). SWI and QSM contrasts are based on a combination of T_2_*-weighted magnitude and filtered phase images (Haacke et al. 2008; Liu et al. 2014). The MT contrast is based on the effect of off-resonance RF pulses on bound and free moving protons (Grossman et al. 1994; McRobbie et al. 2006). Finally the PD contrast reflects the density of the protons (McRobbie et al. 2006). To be able to summarize across the large number of sequence categories no distinction was made between quantitative or qualitative MRI scans (e.g., T_1_ maps versus T_1_ weighted scans or QSM versus SWI).

#### (Near) Isotropic Voxel Size

Isotropic voxels are essential when visualizing small structures, as they have equal biases in all directions when determining the borders. Using anisotropic voxels has the advantage of high in-plane resolution, but determining the border in the z-direction becomes problematic as PVE are increased and can result in measurement biases of subcortical structures (Wonderlick et al. 2009). We determined whether a study acquired isotropic or near isotropic voxels by first calculating the reported voxel volume. For a given volume, the corresponding isotropic voxel dimension was calculated, and compared to the actual acquired voxel size. If the acquired voxel dimensions were within a 10% margin of the isotropic dimensions, the acquired voxel was deemed (near) isotropic, all other voxels were classified as anisotropic.

### Open Access and Interactive Use

All data and code used to analyze and generate the summary figures can be found online (osf.io/fwc2p/, 10.17605/OSF.IO/FWC2P). In addition, a condensed R script is provided which can be used to generate the list of subcortical structures identified with UHF as well to create a summary figure (such as Fig. 6,7 and 8) for a given structure of interest. The R code contains a description of the software requirements as well as instructions for use.

## Results

**Fig. 3:**
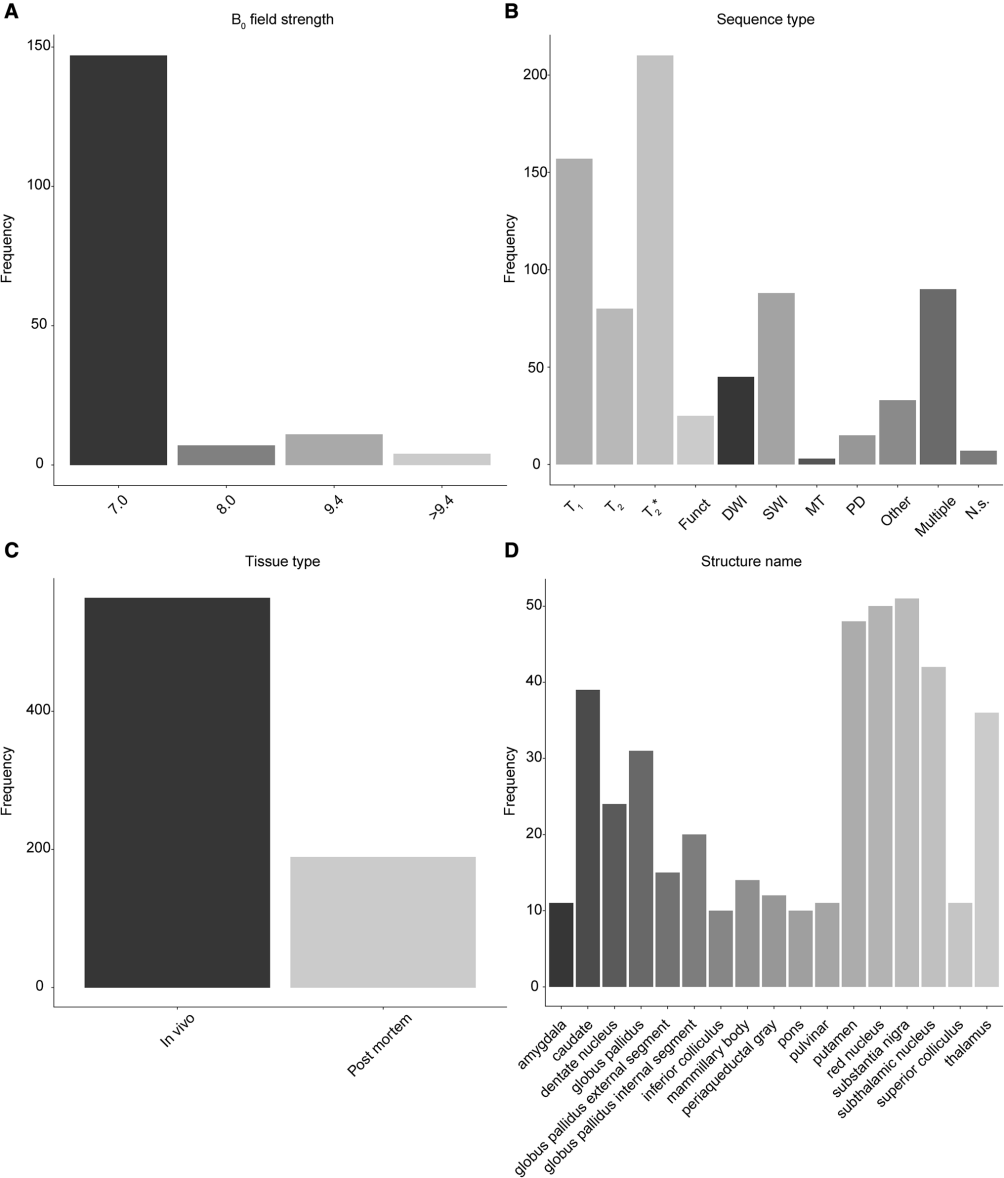
Overview results. **a** The frequency that a MRI scanner with a given B_0_ field strength was used in the 142 studies. **b** The frequency of using a certain MRI sequence type to visualize a subcortical area. **c** Of the 658 cases of identifying a subcortical area, most were done using in vivo samples. **d** The thirteen most frequently reported structures. *Funct* functional MRI sequences that employed functional localizer stimuli, *DWI* diffusion weighted imaging; *SWI* susceptibility weighted imaging, *MT* magnetization transfer; *PD* proton density, *N.s*. not stated

A total of 169 papers were published between 1993 and 2018 that together report the visualization of 163 subcortical structures using 7 T or higher, including both in vivo and post mortem studies. The most frequently employed field strength was 7.0 T (7.0 T: 147 studies; 8.0 T: 7 studies; 9.4 T: 11 studies; 11.7 T: 2 study; 21.1 T: 2 studies; see Fig. 3a). This was expected as the number of 7.0T MRI scanner sites is much larger than that of the higher field strengths (Plantinga et al. 2014). The most frequently employed MRI contrast across the different field strengths and structures were T_2_^*^ based scans, followed by T_1_, SWI, and T_2_ contrasts (see Fig. 3b for the frequency of using a given MRI contrast).

### Demographics

The overall sample size ranged between 1 and 152 participants, with a mean sample size of 18.99 (SD 21.81) and a median of 11 participants across the 169 papers. The in vivo sample size was on average 19.09 (SD 17.93) with a median of 13 participants. The post mortem sample size was on average 15.67 (SD 31.90) with a median of 3.5 specimens. 108 studies included only healthy controls, 13 studies included only patients, 43 studies included both patient and healthy participants, and for 5 studies the participants’ status was not disclosed. The most frequently measured patient groups with UHF MRI were people suffering from Parkinson’s Disease (PD) and Multiple Sclerosis followed by studies that focused on fetal development and or fetal abnormalities. Two out of the six studies that included fetal samples used a wide-bore UHF MRI scanner (see Table 1).

**Table 1:** The publications that identified a subcortical structure with the use of UHF MRI

Publication	Tesla	Vendor	Structure	In vivo/post mortem	Control/patient	Type of patient	N	F/M	Age (sd)
Abduljalil et al. (2003)	8.0	Brucker	cau, gp, put, rn, tha, other	In vivo	Control	–	20	n.s	n.s
Abosch et al. (2010)	7.0	Siemens	gp, gpe, gpi, pul, rn, sn, stn, tha, other	In vivo	Control	–	6	n.s	n.s
Aggarwal et al. (2013)	11.7	Brucker Biospin	other	PM	Control	–	1	1/0	n.s
Al-Helli et al. (2015)	9.4	Varian	stn	PM	Patient	Idiopathic PD	1	0/1	73
Al-Radaideh et al. (2013)	7.0	Phillips	cau, gp, pul, put, other	In vivo	Control	–	20	7/13	34.6 (9.4)
					Patient	Clinically isolated syndrome	19	10/9	26.63 (8.9)
Alarcon et al. (2014)	7.0	Brucker Biospin	cau, gpe, gpi, put, rn, sn, stn, other	PM	Control	–	5	n.s	n.s
Alkemade et al. (2017)	7.0	Siemens	stn	In vivo	Control	–	12	6/6	65 (7.9)
					Patient	PD	12	6/6	68 (9,6)
Augustinack et al. (2014)	7.0	Siemens	mam, rn, sn	PM	Patient	Medically intractable epilepsy	1	0/1	82
Bao et al. (2017)	7.0	Siemens	cau, gp, put, sn, rn	In vivo	Control	–	5	0/5	30–36
Barry et al. (2013)	7.0	Phillips	sn, other	In vivo	Control	–	26	3/23	30.7
Batson et al. (2015)	7.0	Phillips	den, other	In vivo	Control	–	7	¾	31 (n.s.)
Betts et al. (2016)	7.0	Siemens	amy, cau, den, gp, gpe, gpi, put, rn, stn, sn, tha	In vivo	Control	–	40	22/18	47
Beuls et al. (1993)	9.4	Varian	other	PM	n.s	–	5	n.s	n.s
Beuls et al. (2003)	9.4	Varian	pns, other	PM	Patient	Fetal specimen Arnold-Chiari type 2 malformation	1	n.s	20 weeks of GA
					Control	Fetal specimen	1	n.s	21 weeks of GA
Bianciardi et al. (2015)	7.0	Siemens	stn, other	In vivo	Control	–	12	6/6	28 (1)
Bianciardi et al. (2017)	7.0	Siemens	other	In vivo	Control	–	12	6/6	28 (1)
Blazejewska et al. (2013)	7.0	Philips	sn	In vivo	Control	–	2	n.s	39
				PM	Control	–	2	n.s	56
				PM	Patient	PD	1	n.s	75
Blazejewska et al. (2014)	7.0	Philips	rn, sn	In vivo	Control	–	27	n.s	36.4 (8.8)
				In vivo	Patient	Relapsing-remitting MS	14	n.s	42.4 (11.3)
				In vivo	Patient	Clinically Isolated Syndrome	21	n.s	37.2 (8.8
Bourekas and Christoforidis (1999)	8.0	Brucker	cau, gp, gpi, ic, mam, pag, pns,put, rn, sc, sn, tha	In vivo	Control	–	1	1/0	30
Bouvy et al. (2014)	7.0	Philips	put	In vivo	Control	–	13	n.s	18–80
Bouvy et al. (2016)	7.0	Philips	other	In vivo	Control	–	50	30/20	63 (8.5)
Budde et al. (2010)	9.4	Siemens	cau, gp, put, other	In vivo	Control	–	5	n.s	n.s
Budde et al. (2014)	9.4	Siemens	put	In vivo	Control	–	5	1/4	33 (n.s.)
Calamante et al. (2012)	7.0	Siemens	cau, mam, pul, rn, sn, other	In vivo	Control	–	4	2/2	27–31
Chalifoux et al. (2013)	7.0	Siemens	cau, other	In vivo	Patient	Tuberous Sclerosis complex	4	2/2	21.75 (4.35)
Chen et al. (2010)	7.0	Siemens	cau, sn	In vivo	Control	–	1	n.s	n.s
Cho et al. (2008b)	7.0	Siemens	mam, pns, rn, sn, stn, tha, other	In vivo	Control	–	n.s	n.s	early twenties
Cho et al. (2010b)	7.0	Siemens	gpe, gpi, put, sn, stn	In vivo	Control	–	11	n.s	21–30
				In vivo	Patient	PD	1	1/0	48
Cho et al. (2010a)	7.0	Siemens	amy	In vivo	Control	–	13	7/9	42.5 (n.s.)
Cho et al. (2011a)	7.0	Siemens	ic, mam, sc, tha, other	In vivo	Control	–	34	12/22	24.29 (n.s.)
Cho et al. (2011b)	7.0	Siemens	rn, sn	In vivo	Control	–	9	8/1	67.7 (7.4)
				In vivo	Patient	Early PD	8	7/1	58.3 (8.5)
				In vivo	Patient	Late PD	2	1/1	59 (11.3
Cho et al. (2011c)	7.0	Siemens	pul, other	In vivo	Control	–	5	n.s	n.s
Christoforidis et al. (1999)	8.0	Brucker	cau, gp, mam, pul, put, sc, tha, other	In vivo	Control	–	n.s	n.s	n.s
Cosottini et al. (2015)	7.0	GE	rn, other	In vivo	Control	–	13	4/9	54.8 (n.s.)
				In vivo	Patient	PD	14	6/8	57.4 (n.s.)
Cosottini et al. (2014)	7.0	GE	other	In vivo	Control	–	13	4/9	54.7
				PM	Control	–	1	1/0	67
				In vivo	Patient	PD	17	9/8	52.2
Costagli et al. (2015)	7.0	GE	amy, other	In vivo	Control	–	10	3/7	51.7 (n.s.)
Hollander et al. (2014)	7.0	Siemens	stn	In vivo	Control	–	13	6/7	24.38 (2.36)
				PM	Control	–	5	3/2	82.4
de Hollander et al. (2017)	7.0	Siemens	stn	In vivo	Control	–	20	10/10	26 (2.6)
De Martino et al. (2013)	7.0	Siemens	ic, other	In vivo	Control	–	9	4/5	n.s
De Reuck et al. (2014)	7.0	Brucker BioSpin	cau, den, gp, mam, put, rn, sn, stn, tha, other	PM	Control	–	15	2/13	65
				PM	Patient	AD	46	24/22	78
				PM	Patient	Frontotemporal lobar degeneration	37	17/20	68
				PM	Patient	Amyotrophic lateral sclerosis	11	8/3	66
				PM	Patient	Lewy body disease	13	2/11	80
				PM	Patient	PSP	14	10/4	74
				PM	Patient	Vascular dementia	16	9/7	80
De Reuck and Caparros-Lefebvre (2014)	7.0	Brucker BioSpin	den, pns, rn, sn, tha, other	PM	Control	–	11	n.s	n.s
				PM	Patient	PSP	14	n.s	n.s
De Reuck et al. (2015)	7.0	Brucker BioSpin	den	PM	Control	–	16	8/8	68
				PM	Patient	AD	38	17/21	71.82
				PM	Patient	Frontotemporal lobar degeneration	10	4/6	68
				PM	Patient	Amyotrophic lateral sclerosis	9	4/5	65
				PM	Patient	Lewy body disease	10	3/7	82.4
				PM	Patient	PSP	12	8/4	75
				PM	Patient	Vascular dementia	9	6/3	68
De Reuck et al. (2017)	7.0	Brucker BioSpin	put	PM	Control	–	11	3/8	71 (9)
				PM	Patient	Vascular dementia	14	3/11	75 (10)
				PM	Patient	Mixed dementia	24	5/19	76 (11)
Deistung et al. (2013a)	7.0	Siemens	ic, mam, rn, sc, sn, other	In vivo	Control	–	6	2/4	27.3 (3)
Deistung et al. (2013b)	7.0	Siemens	gpe, gpi, mam, pul, put, rn, sc, sn, stn, tha, other	In vivo	Control	–	9	5/9	25.3 (2.8)
Denison et al. (2014)	7.0	Siemens	other	In vivo	Control	–	6	5/1	25–27
Derix et al. (2014)	7.0	Siemens	amy	In vivo	Control	–	6	n.s	24–28
Dezortova et al. (2012)	7.0	Siemens	cau, gp, put	In vivo	Control	–	5	2/3	42 (13.76)
				In vivo	Patient	Panthothenate-kinase associated neurodegeneration	6	4/2	20.47 (7.46)
Di Ieva et al. (2011)	7.0	Siemens	den	In vivo	Control	–	2	n.s	n.s
Diedrichsen et al. (2011)	7.0	Siemens	den, other	In vivo	Control	–	23	14/9	35.1 (13.1)
Dortch et al. (2013)	7.0	Philips	put, tha, other	In vivo	Control	–	13	3/10	22–37
Eapen et al. (2011)	7.0	Philips	mam, rn, sn, other	In vivo	Control	–	10	3/7	20–40
Emir et al. (2012)	7.0	Siemens	pns, put, sn	In vivo	Control	–	12	7/5	54 (8)
				In vivo	Patient	PD	13	6/7	56 (10)
Faull et al. (2015)	7.0	Siemens	amy, cau, gp, put, sn, stn, other	In vivo	Control	–	16	6/10	28(7)
Federau and Gallichan (2016)	7.0	Siemens	amy, cau, gpe, gpi, ic, mam, pag, pul, put, stn, rn, sc, other	In vivo	Control	–	1	0/1	34
Foroutan et al. (2013)	21.1	Brucker BioSpin	gpi, gpe, put, rn, sn	PM	Control	–	3	3/0	70 (4)
				PM	Patient	PSP	6	6/0	76 (6)
Forstmann et al. (2010)	7.0	Siemens	stn	In vivo	Control	–	9	6/3	24.5 (2.1)
Forstmann et al. (2012)	7.0	Siemens	stn	In vivo	Control	–	13	6/7	24.38 (2.36)
Forstmann et al. (2014)	7.0	Siemens	cau, gpe, gpi, put, rn, sn, stn, tha, other	In vivo	Control	–	54	25/29	39.72 (n.s.)
Forstmann et al. (2017b)	7.0	n.s	gp, stn, other	In vivo	Patien	PD	1	0/1	57
Fritzsch et al. (2014)	7.0	Siemens	gp, put, rn, sn, other	In vivo	Control	–	10	5/5	44 (n.s.)
				In vivo	Patient	Wilson’s Disease	11	6/5	41 (n.s.)
Frosini et al. (2017)	7.0	GE	other	In vivo	Control	–	10	3/7	65.2 (5.1)
				In vivo	Patient	MSA	6	n.s	64.5 (7.64)
				In vivo	Patient	PSP	5	n.s	71.4 (8.82)
				In vivo	Patient	CBD	4	n.s	69.8 (4.57)
Fujioka et al. (2011)	21.1	Brucker BioSpin	gpe, gpi, put	PM	Control	–	1	0/1	87
				PM	Patient	Diffuse Lewy body disease	1	0/1	81
Ghaznawi et al. (2017)	7.0	Philips	cau	In vivo	Patient	Systematic atherosclerotic disease	90	17/73	68 (8)
Gizewski et al. (2007)	7.0	Siemens	tha	In vivo	Control	–	9	2/7	31 (n.s.)
Gizewski et al. (2013)	7.0	Siemens	pag, other	In vivo	Control	–	8	5/3	31 (n.s.)
Gorka et al. (2017)	7.0	Siemens	other	In vivo	Control	–	27	14/13	27.3 (6)
Grabner et al. (2014)	7.0	Siemens	den	In vivo	Control	–	8	n.s	n.s
Hammond et al. (2008a)	7.0	GE	cau, gpe, gpi, pag, pns, put, rn, sn, tha	In vivo	Control	–	12	5/7	36.9 (n.s.)
				In vivo	Patient	MS	10	3/3	43.6 (n.s.)
				In vivo	Patient	Brain tumors	25	10/15	48.6 (n.s.)
Hammond et al. (2008b)	7.0	GE	cau, gp, put, tha	In vivo	Control	–	13	8/5	40.15 (14.19)
				In vivo	Patient	Relapse remitting MS	19	16/6	42.32 (12.9)
Kanowski et al. (2014)	7.0	Siemens	other	In vivo	Control	–	5	3/2	21–28
Keren et al. (2015)	7.0	Brucker	other	PM	Patient	AD	7	4/3	76.4 (9.5)
Kerl et al. (2012)	7.0	Siemens	rn, sn, stn, other	In vivo	Control	–	9	4/5	25 (n.s.)
Kerl (2013)	7.0	Siemens	gp, rn, sn, other	In vivo	Control	–	9	4/5	25 (n.s.)
Keuken et al. (2013)	7.0	Siemens	stn	In vivo	Control	–	31	15/16	45.93 (n.s.)
Keuken et al. (2014)	7.0	Siemens	stn	In vivo	Control	–	30	14/16	24.2 (2.4)
Keuken et al. (2015)	7.0	Siemens	gpe, gpi, rn, sn, stn, other	In vivo	Control	–	15	9/6	23.7 (1.58)
Keuken et al. (2017)	7.0	Siemens	gpe, gpi, pag, rn, sn, stn, other	In vivo	Control	–	53	21/31	39.72 (n.s.)
Khabipova et al. (2015)	7.0	Siemens	cau, gp, put, rn, sn	In vivo	Control	–	3	1/2	30 (6)
				In vivo	Patient	MS	1	n.s	n.s
Kim et al. (2011)	7.0	n.s	other	In vivo	Control	–	20	6/14	22–30
Kim et al. (2014)	7.0	n.s	cau, gpe, gpi, put, sn, stn, tha	In vivo	n.s	–	5	n.s	n.s
Kim et al. (2015a)	7.0	n.s	ic, pns, sc, tha, other	In vivo	Control	–	16	4/12	30 (7.9
Kim et al. (2015b)	7.0	n.s	pul, other	In vivo	Control	–	15	5/10	30.5
				In vivo	Patient	Schizophrenia	12	3/9	29.7
Kim et al. (2016)	7.0	Siemens	sn	In vivo	Control	–	26	15/11	49.5 (12.6)
				In vivo	Patient	PD	30	15/15	51.0 (9.6)
				In vivo	Patient	MSA	7	6/1	55.3 (6.1)
				In vivo	Patient	PSP	3	0/3	71.0 (4.6)
Kim et al. (2017a)	7.0	n.s	other	In vivo	Control	–	18	5/13	32.6 (12)
				In vivo	Patient	Schizophrenia	19	7/12	30.7 (7.9)
Kim et al. (2017b)	7.0	Siemens	cau, put, sn, stn, other	In vivo	Control	–	n.s	n.s	n.s
Kirov et al. (2013)	7.0	Siemens	rn	In vivo	Control	–	15	7/8	35.6 (9.4)
	7.0			In vivo	Patient	Schizophrenia	16	6/10	40.7 (10.6)
Kollia et al. (2009)	7.0	Siemens	den	In vivo	Patient	MS	12	8/4	32 (n.s.)
Küper et al. (2011a)	7.0	Siemens	den	In vivo	Control	–	23	0/23	28.1 (6.3)
Küper et al. (2011b)	7.0	Siemens	den	In vivo	Control	–	23	0/23	28.1 (6.3)
Küper et al. (2013)	7.0	Siemens	den	In vivo	Control	–	19	7/12	26.6 (3.8)
Kwon et al. (2012)	7.0	Siemens	rn, sn, stn	In vivo	Control	–	10	9/1	59.7 (5.1)
				In vivo	Patient	PD	10	7/3	60 (7.2)
Lee et al. (2014)	7.0	Siemens	other	In vivo	Control	–	18	10/8	45.2 (10.9)
				In vivo	Patient	Primary open-angle glaucoma	18	10/8	47.6 (13.3)
Lenglet et al. (2012)	7.0	Siemens	cau, gpe, gpi, put, sn, stn, tha	In vivo	Control	–	4	n.s	23–57
Liem et al. (2012)	7.0	Philips	gp, put, tha, other	In vivo	Control	–	18	8/10	45.8 (12.8)
				In vivo	Patient	NOTCH3 mutation carriers	25	13/12	46.5 (12.2)
				PM	Patient	NOTCH3 mutation carriers	3	2/1	60.67 (3.06)
Liu et al. (2011)	7.0	Brucker	den	PM	Control	Fetal specimen	40	n.s	14–22 weeks GA
Lotfipour et al. (2011)	7.0	Philips	rn, sn, other	In vivo	Control	–	11	7/4	59.13 (8.59)
				In vivo	Patient	PD	9	5/4	64.67 (13.28)
Makris et al. (2013a)	7.0	n.s	other	PM	Control	–	2	0/2	40 (15.57)
Marques et al. (2010)	7.0	Siemens	den	In vivo	Control	–	3	1/2	30 (n.s.)
Marques and Gruetter (2013)	7.0	Siemens	cau, put, other	In vivo	control	–	7	n.s	26.29 (n.s.)
Massey et al. (2012)	9.4	Varian	gp, gpi, mam, pul, rn, sc, sn, stn, tha, other	PM	Control	–	8	4/4	77.34 (17.64)
Meijer et al. (2016)	11.7	Brucker	rn, other	PM	Control	–	2	2/0	80 (5.66)
					Patient	PD	2	1/1	78.5 (3.53)
Meng et al. (2012)	7.0	Brucker	cau, other	PM	Control	Fetal specimen	69	n.s	12–22 weeks GA
Mestres-Missé et al. (2014)	7.0	Siemens	other	In vivo	Control	–	23	11/12	26 (3)
Miller et al. (2015)	7.0	Philips	amy	In vivo	Control	–	1	0/1	42
Mitsumori et al. (2011)	7.0	Siemens	cau, gp, put, tha	In vivo	Control	–	6	0/6	49.3 (8)
Moenninghoff et al. (2010)	7.0	Siemens	den	In vivo	Patient	Lhermitte–Duclos disease	1	0/1	46
Moerel et al. (2015)	7.0	Siemens	other	In vivo	Control	–	6	5/1	25 (1.7)
Mollink et al. (2016)	7.0	Siemens	den, tha	PM	Control	–	1	1/0	87
Novak et al. (2001a)	8.0	Brucker	ic, pag, pns, rn, sc, sn, other	In vivo	Control	–	5	2/3	34–46
Novak et al. (2001b)	8.0	Brucker	cau, gp	In vivo	Control	–	11	n.s	37–59
				In vivo	Patient	Hypertensive	6	n.s	37–59
O’Brien et al. (2014)	7.0	Siemens	other	In vivo	Control	–	8	2/6	29 (4.1)
				In vivo	Patient	Epilepsy	2	n.s	n.s
Plantinga et al. (2016a)	7.0	Siemens	gpe, gpi, stn, other	PM	Control	–	1	n.s	70–95
Plantinga et al. (2016b)	7.0	Siemens	stn	In vivo	Patient	PD	17	5/12	62
Peters et al. (2007)	7.0	Philips	cau, put	In vivo	Control	–	6	n.s	37 (11)
Rijkers et al. (2007)	9.4	Varian unity	pag, pul, rn, sc, sn, stn, other	PM	n.s	–	1	n.s	n.s
Robitaille and Kangarlu (1999)	8.0	Brucker	mam, rn, other	In vivo	n.s		n.s	n.s	n.s
Romanzetti et al. (2014)	9.4	Siemens	tha	In vivo	Control	–	19	3/16	36 (4)
Rooney et al. (2007)	7.0	n.s	cau, gp, put, tha	In vivo	Control	–	3	0/3	32–59
de Rotte et al. (2014)	7.0	Philips	other	In vivo	Control	–	10	6/4	25 (n.s.)
				In vivo	Patient	Micro adenoma	5	n.s	35.2 (12.40)
de Rotte et al. (2015)	7.0	Philips	other	In vivo	Patient	Cushing disease	16	n.s	n.s
Rudko et al. (2014)	7.0	Agilent	cau, gp, put, tha	In vivo	Control	–	15	12/3	36.4 (6.42)
				In vivo	Patient	MS	25	18/7	37.3 (6.1)
Satpute et al. (2013)	7.0	Siemens	pag	In vivo	Control	–	11	6/5	20–35
Schäfer et al. (2009)	7.0	Philips	rn, sn, stn	In vivo	Control	–	n.s	n.s	n.s
Schäfer et al. (2012)	7.0	Siemens	rn, sn	In vivo	Control	–	8	3/5	22–28
Schindler et al. (2013)	7.0	Siemens	gpi, mam, sn, stn, tha, other	In vivo	Control	–	10	8/2	38.5 (13.6)
Schindler et al. (2017)	7.0	Siemens	other	In vivo	Control	–	84	51/33	39 (13)
Schmidt et al. (2017a)	7.0	Siemens	other	In vivo	Control	–	20	12/8	36.45 (13.16)
				In vivo	Patient	Unmedicated MDD	20	12/8	36.20 (12.83)
				In vivo	Patient	Medicated MDD	20	13/7	40.60 (12.11)
Schmidt et al. (2017b)	7.0	Siemens	other	In vivo	Control	–	13	5/8	46.7 (12.5)
Schreiner et al. (2014)	7.0	Philips	amy, cau, gp, put, tha, other	In vivo	Control	–	14	6/8	68.43 (5.3)
Shmueli et al. (2009)	7.0	GE	put, rn, sn	In vivo	Control	–	1	n.s	n.s
Sladky et al. (2013)	7.0	Siemens	amy	In vivo	Control	–	15	6/9	29.54 (6.65)
Solano-Castiella et al. (2011)	7.0	Siemens	amy, other	In vivo	Control	–	9	n.s	21–29
Solbach et al. (2014)	7.0	Siemens	den	In vivo	Control	–	14	7/7	38.1 (7.7)
				In vivo	Patient	Friedreich’s ataxia	14	8/6	38.1 (8.5)
Soria et al. (2011)	7.0	Brucker	ic, pag, rn, other	PM	Control		3	n.s	n.s
Stefanescu et al. (2013)	7.0	Siemens	den	In vivo	Control	–	19	9/10	26.5 (3.5)
Stefanescu et al. (2015)	7.0	Siemens	den	In vivo	Control	–	23	10/13	46.39 (15.82)
				In vivo	Patient	SCA6	12	5/7	57.75 (12.06)
				In vivo	Patient	Friedreich’s ataxia	12	7/5	39.08 (12.87)
				In vivo	Patient	SCA3	10	3/7	47.2 (10.58)
Strotmann et al. (2013b)	7.0	Siemens	other	PM	Control	–	1	1/0	65
Strotmann et al. (2013a)	7.0	Siemens	other	In vivo	Control	–	3	n.s	n.s
				PM	Control	–	1	1/0	65
Stüber et al. (2014)	7.0	Siemens	sn, stn	PM	n.s	–	1	n.s	n.s
Tang et al. (2014)	7.0	Philips	other	In vivo	Control	–	1	0/1	42
Thayyil et al. (2009)	9.4	Varian	tha	PM	Patient	Fetal specimen	17	n.s	less than 22 weeks of GA
Thomas et al. (2008)	7.0	Philips	amy	In vivo	Control	–	6	0/6	32 (n.s.)
Thulborn et al. (2015)	9.4	GE	tha, other	In vivo	Control	–	49	26/23	48 (19)
Thürling et al. (2011)	7.0	Siemens	den	In vivo	Control	–	17	0/17	27.4 (6.4)
Thürling et al. (2012)	7.0	Siemens	den	In vivo	Control	–	21	10/11	25.5 (3.9)
				In vivo	Control	–	23	8/15	27 (3.8)
Thürling et al. (2015)	7.0	Siemens	den, other	In vivo	Control	–	24	11/13	31.8 (6.4)
Tourdias et al. (2014)	7.0	GE	pul, rn, stn, other	In vivo	Control	–	6	1/5	31.2 (n.s.)
Trampel et al. (2013)	7.0	Siemens	other	In vivo	n.s	–	n.s	n.s	n.s
Truong et al. (2006b)	8.0	Brucker	gp, put, rn, sn	In vivo	Control	–	2	2/0	34 (0)
				PM	Patient	Various neuropathologies	4	2/2	72–81
van Bergen et al. (2016)	7.0	Philips	sn, rn	In vivo	Control	–	16	8/8	43.3 (11.7)
				In vivo	Patient	Premanifest Huntington Disease	15	5/10	42.4 (8.7)
van den Bogaard et al. (2011)	7.0	Philips	cau, put, tha, other	In vivo	Control	–	18	9/9	47.7 (7.4)
				In vivo	Patient	Premanifest Huntington Disease	14	8/6	42.9 (11)
				In vivo	Patient	Manifest Huntington Disease	12	7/5	48.6 (7)
Verma et al. (2013)	7.0	Siemens	other	In vivo	Control	–	2	n.s	38.5 (10.61)
Visser et al. (2016a)	7.0	Siemens	cau, gp, put	In vivo	Control	–	54	25/29	39.72 (n.s.)
Visser et al. (2016b)	7.0	Siemens	sn, stn, rn	In vivo	Control	–	54	25/29	39.72 (n.s.)
Wang et al. (2016)	7.0	Siemens	other	In vivo	Control	–	53	21/31	39.72 (n.s.)
Wargo and Gore (2013)	7.0	Philips	pns, put, rn, tha	In vivo	Control	–	8	4/4	20–54
Weiss et al. (2015)	7.0	Siemens	stn	PM	Control	–	4	3/1	66.75 (19.48)
Wharton et al. (2010)	7.0	Philips	rn, sn	In vivo	Control	–	3	n.s	n.s
Wharton and Bowtell (2010)	7.0	Philips	cau, gp, put, rn, sn, tha	In vivo	Control	–	5	0/5	25–30
Wright et al. (2008)	7.0	Philips	cau, put	In vivo	Control	–	4	1//3	36.5 (8.5)
Yang et al. (2013)	7.0	Siemens	den	PM	Control	–	2	2/0	74.5 (2.12)
Yao et al. (2009)	7.0	GE	cau, gp, put, tha	In vivo	Control	–	9	4/5	31 (5)
				PM	Control	–	2	0/2	68 (2)
Zeineh et al. (2014)	7.0	GE	rn, sn, stn	In vivo	Control	–	6	n.s	n.s
Zhang et al. (2011)	7.0	Brucker	cau, other	PM	Control	Fetal specimen	20	10/10	20 weeks of GA
Zielman et al. (2014)	7.0	Philips	pns, other	In vivo	Control	–	19	12/7	38.5 (12.1)
				In vivo	Patient	Hemiplegic migraine	18	11/7	38.1 (14.4)
Zrinzo et al. (2011)	9.4	Varian	pag	PM	Control	–	1	0/1	68
Zwanenburg et al. (2008)	7.0	Philips	gp, put, tha, other	In vivo	Control	–	7	1/6	26 (10)
Zwanenburg et al. (2009)	7.0	Philips	stn	In vivo	Control	–	5	1/5	24 (4)

*n.s*. Not stated, *PM* post mortem, *PD* Parkinson’s Disease, *AD* Alzheimer Disease, *MDD* major depressive disorder, *MS* multiple sclerosis, *PSP* progressive supranuclear palsy, *GA* gestation, *MSA* multiple system atrophy, *CBD* corticobasal degeneration, The seventeen most frequently reported structures were: amy: amygdala, *cau* caudate, *den* dentate nucleus, *gp* globus pallidus, *gpe* globus pallidus external segment, *gpi* globus pallidus internal segment, *ic* inferior collicus, *mam* mammillary body, *pag* periagueductal gray, *pns* pons, *pul* pulvinar, *put* putamen, *rn* red nucleus, *sn* substantia nigra, *stn* subthalamic nucleus, *sc* superior colliculus, *tha* thalamus. The remaining structures are indicated with the label other

### Subcortical Structures

The frequency with which a structure was reported ranged between 1 and 51, with a mean reported frequency of 4.62 (SD 8.88) and a median of 1. There are 55 UHF MRI studies that only reported a single structure, whereas for 83 structures there was only a single UHF MRI study that visualized that specific structure [e.g., for the locus coeruleus (Keren et al. 2015); the field of Forel (Massey et al. 2012); and a number of thalamic sub-nuclei such as the magno- and parvocellular part of the lateral geniculate nucleus (Denison et al. 2014)]. The SN was reported most frequently (51 reports), closely followed by the red nucleus (50 reports) and putamen (48 reports; see Fig. 3d for the seventeen most frequently reported structures).

### Identification Versus Parcellation

Of the 753 reports across the 169 papers, there were 344 reports where the authors (partially) parcellated a subcortical structure. This was either done by manual parcellation (208 reports), placing a ROI in a visually identified area (51 reports), semi-automatic procedures (22 reports), fully automatic procedures (26 reports), using a functional localizer (5 reports), or otherwise parcellated in a way that was unclear from the manuscript (32 reports). Overall, regardless of method, the most frequently parcellated structure was the putamen (31 reports) whereas the STN was the most frequently manually parcellated structure (21 reports).

**Fig. 4:**
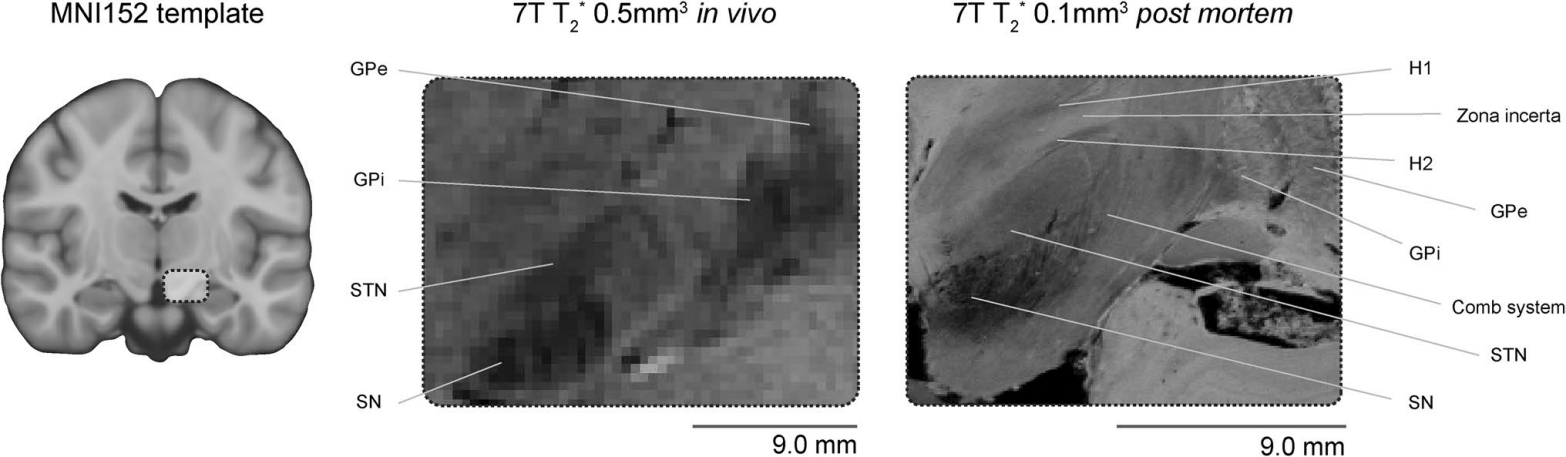
In vivo versus post mortem comparison. The left panel shows the MNI152 template with a highlighted subcortical region. The middle panel highlights this subcortical region using a 7T in vivo 0.5 mm isotropic resolution T_2_*-weighted structural scan where the globus pallidus externa (GPe), globus pallidus interna (GPi), STN and SN can be visualized. The right panel illustrates a similar region in a post mortem sample scanned with a 0.1 mm isotropic resolution T_2_*-weighted scan where a number of subcortical areas can be identified which are not clearly visible in the in vivo scans such as the fields of Forel (H1, H2), zona incerta and the comb system. Image is adapted from (Forstmann et al. 2017a)

Of the 344 parcellated reports there were 75 structures parcellated in vivo, and 36 structures parcellated using post mortem samples. There is an overlap of 17 structures that are parcellated in both in vivo and post mortem data (see Fig. 4 for a comparison between the image quality achievable with in vivo versus post mortem scanning).

**Fig. 5:**
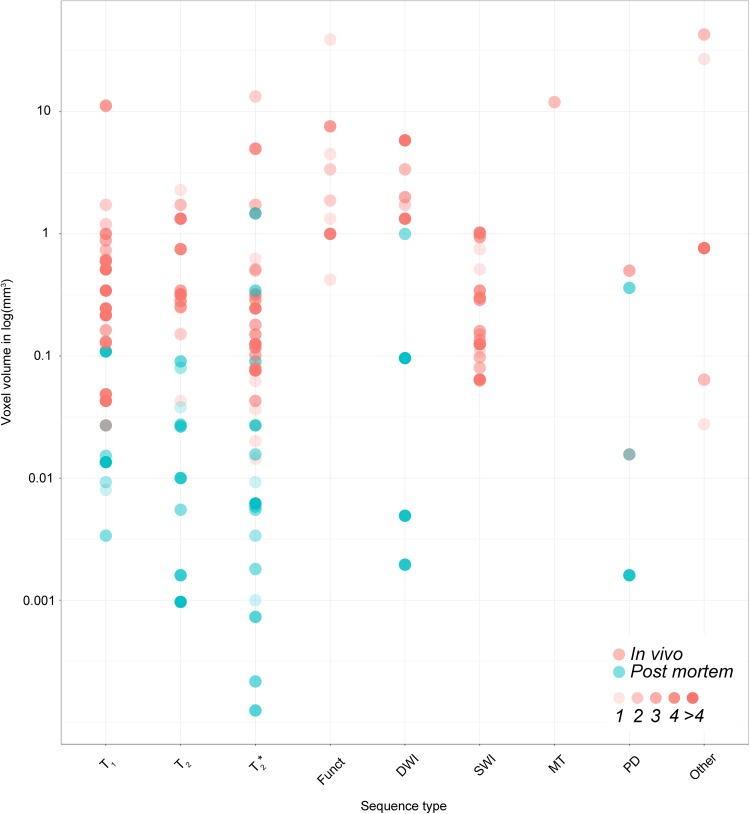
Voxel volume for the different MRI sequences. Each dot represents the voxel volume used to visualize a subcortical structure across the 169 studies. The in vivo samples are displayed in red, whereas the post mortem samples are shown in blue. The color intensity corresponds to the number of studies using the same voxel volume. *Funct* functional MRI sequences that employed functional localizer stimuli, *DWI* diffusion weighted imaging, *SWI* susceptibility weighted imaging, *MT* magnetization transfer, *PD* proton density, *N.s*. not stated, *PD* (patient type) Parkinson’s disease, *MS* multiple sclerosis

Structures which were only parcellated using post mortem data include a number of small structures in the lower brainstem such as the abducens nucleus, primary olivary nucleus, cuneate nucleus, a number of sub-nuclei of the hypothalamus, and the claustrum. That the claustrum has never been parcellated in vivo was somewhat surprising as it is a relatively large structure, medial to the striatum. A potential explanation why such small structures in the brainstem are only parcellated using post mortem data is the employed voxel volume (see Fig. 5 for an overview of voxel volumes used per MRI sequence and sample type). One of the benefits of post mortem scanning is the possibility to employ longer scan times in the absence of motion, which allows for the acquisition of smaller voxels, and/or the possibility of scanning a smaller sample at higher fields than available in vivo [e.g., 0.05 mm isotropic voxels with an acquisition of 4.3 h using 21.1T (Foroutan et al. 2013) or 0.09 mm isotropic voxels with an acquisition of 10.5 h using 7.0T (Makris et al. 2013b)].

### Voxel Volume and Isotropic Voxels

The voxel volume across the different structural MRI contrasts including the DWI scans for the in vivo scans ranged between 0.0144 and 42.875 mm^3^, with a mean volume of 1.09 mm^3^ (SD 3.71 mm^3^) and a median of 0.245 mm^3^. The voxel volume for the functional MRI contrasts for the in vivo scans ranged between 0.422 and 39.051 mm^3^, with a mean volume of 4.50 mm^3^ (SD 7.72 mm^3^) and a median of 1.33 mm^3^. For the post mortem scans the volume varied between 0.000125 and 1.47 mm^3^ with a mean voxel volume of 0.075 mm^3^ (SD 0.23 mm^3^) and a median of 0.01 mm^3^. See Fig. 5 for an overview of voxel volumes used per MRI sequence.

Of all the structures that were identified using a T_1_ based contrast, 128 reports of structures were achieved using isotropic or near isotropic voxels, and 83 reports were based on anisotropic voxels. For the T_2_ based contrasts sequences, 26 reports were based on isotropic voxels, and 90 reports were based on anisotropic voxels. Using a T_2_* sequence, 114 reports were based on isotropic voxels, whereas 138 reports were not. For the functional sequences, all 25 reports were based on isotropic voxels. The DWI sequence resulted in 60 reports using isotropic voxels and 27 reports using anisotropic voxels. SWI sequences that were used to identify structures were isotropic in 82 cases and in 21 cases anisotropic. All three reports that identified a structure using an MT based sequence were based on anisotropic voxels. The PD sequences that were used to identify structures were isotropic for 6 reports and 18 reports were based on anisotropic voxels.

### Volumetric Reports

With a total of 51 reports, the SN is the most frequently visualized structure, of which only 9 papers provide an explicit volume estimate (see Table 2). For the STN, directly adjacent to the SN, there are 42 reports, of which there are 12 reports that provide a volume estimate. There is substantial variability in volume estimates for both structures. For the SN, volumes range between 224.75 and 1300 mm^3^. For the STN the volumes range between 37.32 and 223 mm^3^. The volumes are based on a range of different MRI contrasts and parcellation methods, such as automatic segmentations or the conjunction of two manual raters. This variability in methods makes it problematic to provide a summary of volume estimates and whether there is a systematic difference due to acquisition technique.

**Table 2:** SN and STN volume estimates

Publication	Structure	Volume estimate	Population	Segmentation method	MRI contrast	Voxel dimension
Bianciardi et al. (2015)	SN	490 mm^3^	Control	Semi-automatic	FA & T_2_	1.1 × 1.1 × 1.1
Chen et al. (2010)	SN	79 mm^2a^	Control	Manual	T_2_*	0.25 × 0.25 × 2.0
Eapen et al. (2011)	SN	725.7 mm^3^	Control	Automatic	T_2_ (Hybrid Echo)	0.4 × 0.4 × 2.0
	SN	753.1 mm^3^	Control	Automatic	T_2_*	0.4 × 0.4 × 2.0
Keuken et al. (2014)	SN	224.75 mm^3^	Control	Conj. masks of two manual raters	T_2_*	0.5 × 0.5 × 0.5
Keuken et al. (2017)	SN	270.36 mm^3^	Control	Conj. masks of two manual raters	T_2_*	0.5 × 0.5 × 0.5
Kwon et al. (2012)	SN	270.63 mm^3^	Control	Masks of two manual raters	T_2_*	0.35 × 0.35 × 0.35
	SN	310.68 mm^3^	PD	Masks of two manual raters	T_2_*	0.35 × 0.35 × 0.35
Plantinga et al. (2016a)	SN	281.4 mm^3b^	PM Control	Manual	T_2_*	0.3 × 0.3 × 0.3
Lenglet et al. (2012)	SN	586 mm^3c^	Control	Manual masks	T_2_ + SWI	0.4 × 0.4 × 2.0
van Bergen et al. (2016)	SN	1300 mm^3^	Control	Semi-automatic	SWI	1.0 × 1.0 × 1.0
		1300 mm^3^	Premanifest HD	Semi-automatic	SWI	1.0 × 1.0 × 1.0
Alkemade et al. (2017)	STN	82.34 mm^3^	Control	Conj. masks of two manual raters	QSM	0.5 × 0.5 × 0.6
	STN	76.8 mm^3^	PD	Conj. masks of two manual raters	QSM	0.6 × 0.6 × 0.8
Bianciardi et al. (2015)	STN	163.5 mm^3^	Control	Semi-automatic	FA & T_2_	1.1 × 1.1 × 1.1
Keuken et al. (2013)	STN	63.13 mm^3^	Control	Conj. masks of two manual raters	T_2_*	0.5 × 0.5 × 0.6
Keuken et al. (2014)	STN	56.17 mm^3^	Control	Conj. masks of two manual raters	T_2_*	0.5 × 0.5 × 0.5
Keuken et al. (2015)	STN	62.25 mm^3^	Control	Conj. masks of two manual raters	T_2_*	0.5 × 0.5 × 0.5
Keuken et al. (2017)	STN	37.32 mm^3^	Control	Conj. masks of two manual raters	T_2_*	0.5 × 0.5 × 0.5
Lenglet et al. (2012)	STN	223.5 mm^3 c^	Control	Manual mask	T_2_ + SWI	0.4 × 0.4 × 2.0
Massey et al. (2012)	STN	198 mm^3^	PM Control	Manual mask	T_2_*	0.18 × 0.18 × 0.18
Plantinga et al. (2016a)	STN	100.5 mm^3^	PM Control	Manual mask	T_2_*	0.3 × 0.3 × 0.3
Plantinga et al. (2016b)	STN	125.4 mm^3^	PD	Manual mask	T_2_	0.39 × 1.0 × 0.39
Schäfer et al. (2012)	STN	48 mm^3^	Control	Masks of two manual raters	T_2_*	0.5 × 0.5 × 0.6
Weiss et al. (2015)	STN	109 mm^3^	PM Control	Conj. masks of two manual raters	T_2_*	0.1 × 0.1 × 0.1

*a* Single slice, *b* SNc and SNr combined, *c* extracted using webplot digitizer, *PD* Parkinson Disease, *PM* post mortem, *FA* Fractional Anisotropy, *n.s*. not stated, *SWI* susceptibility weighted imaging, *Conj* conjunction. Voxel dimension is in mm

#### MRI Contrasts for Visualizing the SN, STN, and Thalamus

**Fig. 6:**
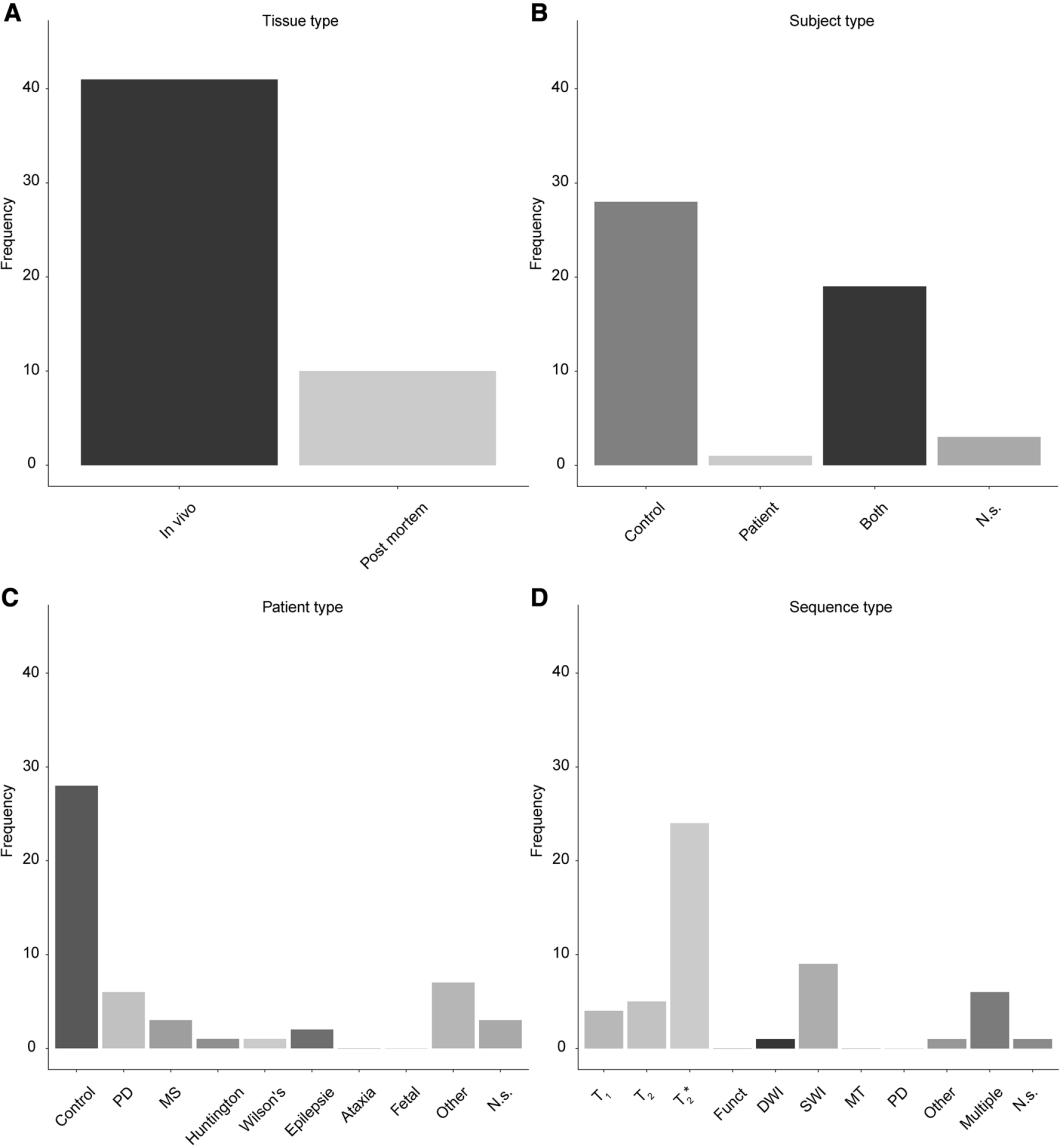
Overview of the use of UHF MRI for visualizing the substantia nigra. **a** Of the 51 studies that identified the SN, most were done using in vivo samples. **b** Most studies only used healthy controls, whereas a substantial number also included patients. **c** The studies that included a clinical group mainly focused on Parkinson’s Disease patients or abnormal fetal developments. **d** The frequency of using a certain MRI sequence type to visualize the SN. The most frequently used contrast was a T_2_* type of sequence. *Funct* functional MRI sequences that employed functional localizer stimuli, *DWI* diffusion weighted imaging, *SWI* susceptibility weighted imaging, *MT* magnetization transfer, *PD* proton density, *N.s*. not stated, *PD* (patient type) Parkinson’s Disease, *MS* multiple sclerosis

It is interesting to note the variability in MRI contrasts used to visualize a number of subcortical structures. For the SN by far the most commonly used contrast is a T_2_* based sequence followed by SWI contrasts (Fig. 6d). Given that the SN contains relatively large amounts of iron, which increases the magnetic susceptibility, it is not surprising that T_2_* and SWI seem to be the contrasts of choice (Hallgren and Sourander 1958; Chavhan et al. 2009). In terms of demographics, the SN is regularly visualized in PD patients, which is expected due to the underlying pathology occurring in the SN in PD (Fig. 6c).

**Fig. 7:**
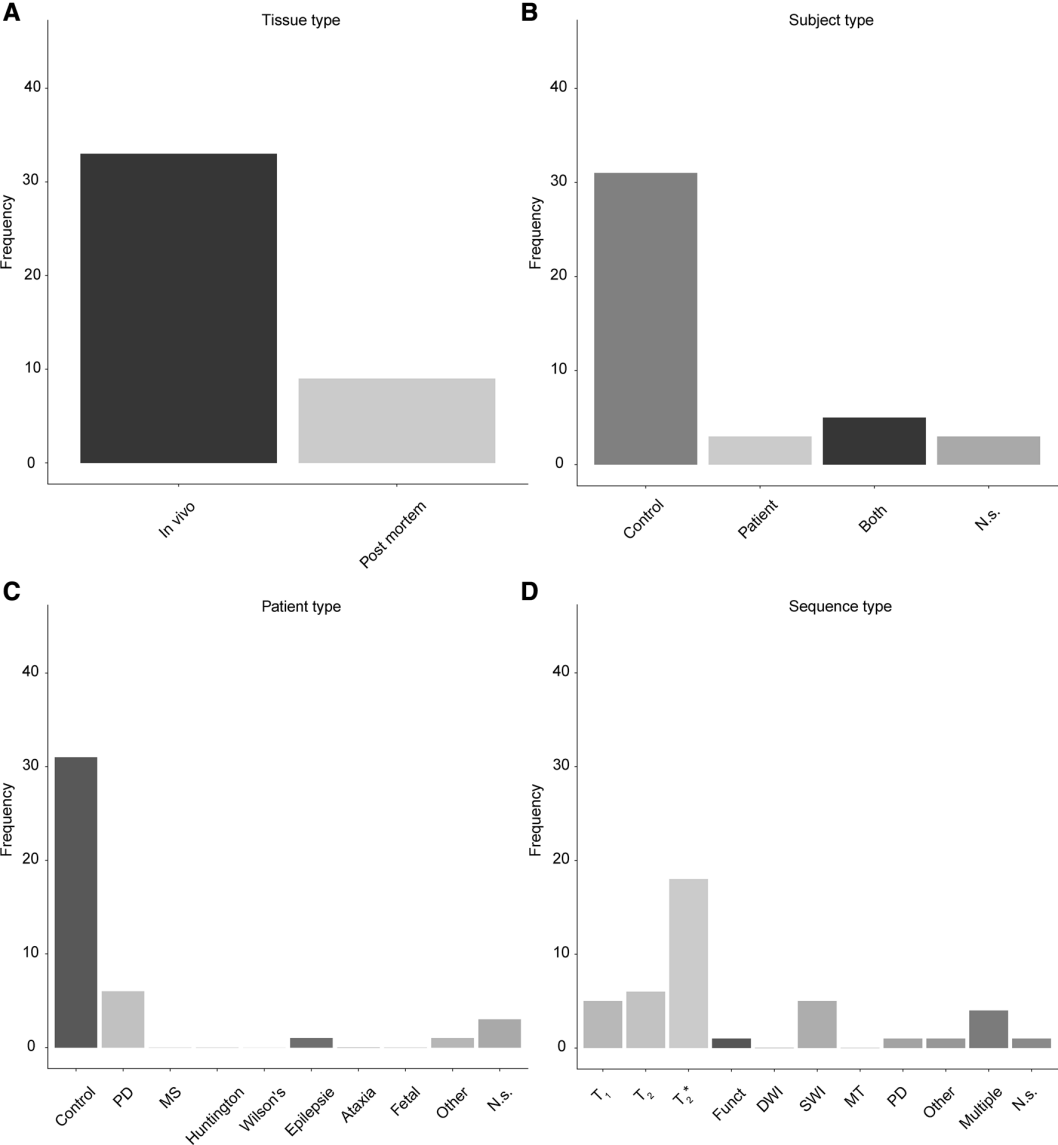
Overview of the use of UHF MRI for visualizing the subthalamic nucleus. **a** Of the 42 studies that identified the STN, most were done using in vivo samples. **b** Most studies only used healthy controls. Compared to the SN there were substantially fewer studies that also included patients. **c** The studies that included a clinical group mainly focused on Parkinson’s Disease patients or abnormal fetal developments. **d** The frequency of using a certain MRI sequence type to visualize the STN. The most frequently used contrast was a T_2_* type of sequence. *Funct* functional MRI sequences that employed functional localizer stimuli, *DWI* diffusion weighted imaging, *SWI* susceptibility weighted imaging, *MT* magnetization transfer, *PD* proton density, *N.s*. not stated, *PD* (patient type) Parkinson’s Disease, *MS* multiple sclerosis

Another structure which is implicated in the pathophysiology of PD is the STN, a structure also high in iron content and located directly adjacent to the SN. As with the SN, the most frequently used contrast mechanism to visualize the STN is T_2_* (Fig. 7d). The ratio for identification versus parcellation of the STN is larger than for the SN. Additionally, the STN is more commonly visualized in the healthy population, compared to the SN which included relatively more clinical groups (Fig. 6c versus Fig. 7c).

**Fig. 8:**
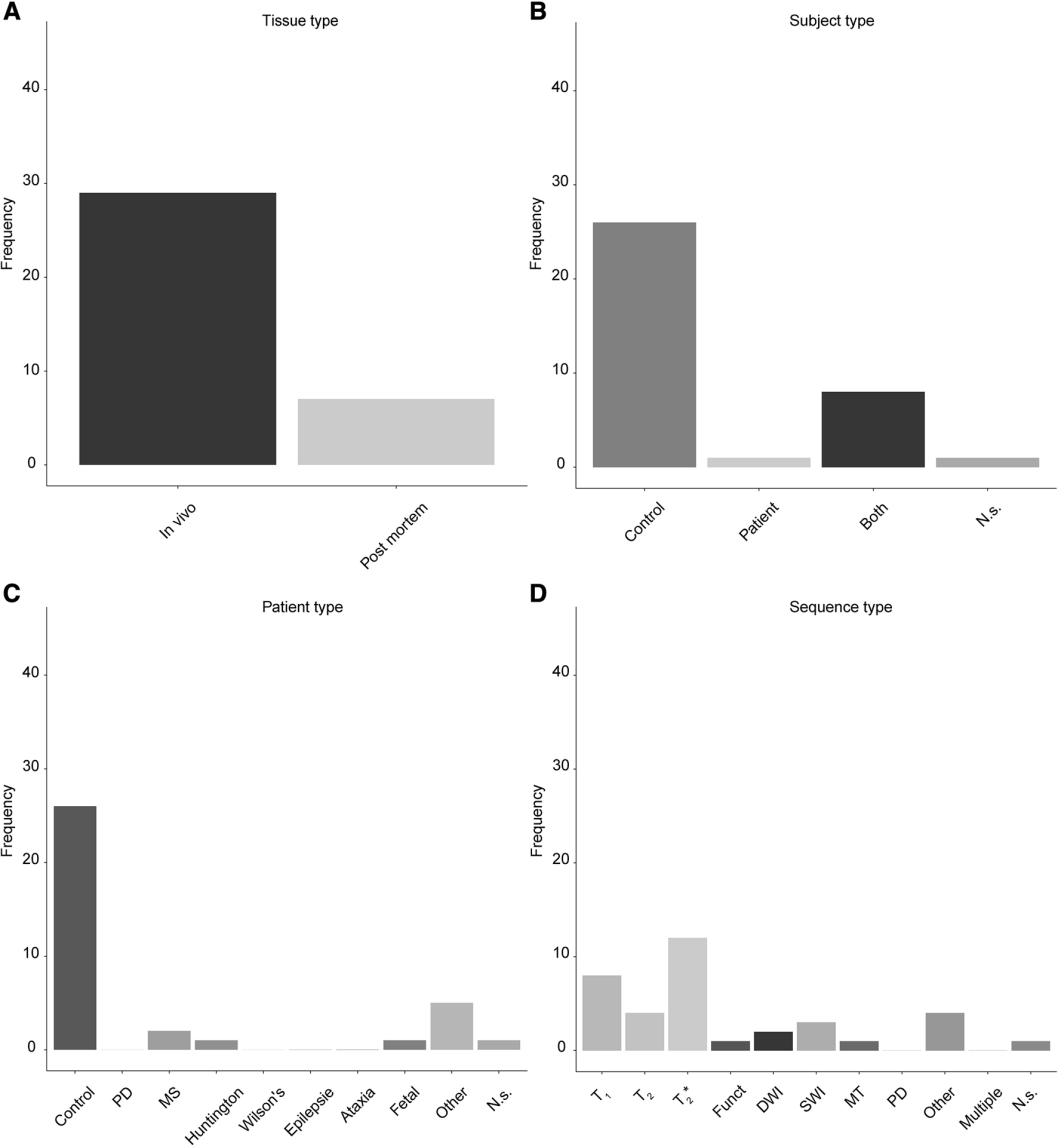
Overview of the use of UHF MRI for visualizing the thalamus. **a** Of the 36 studies that identified the Th, most were done using in vivo samples. **b** Most studies only used healthy controls. Compared to the SN, there were substantially fewer studies that also included patients. **c** The studies that included a clinical group mainly focused on abnormal fetal developments. **d** The frequency of using a certain MRI sequence type to visualize the Th. The most frequently used contrast was a T_2_* type of sequence, followed closely by T_1_ type of sequences. *Funct* functional MRI sequences that employed functional localizer stimuli, *DWI* diffusion weighted imaging, *SWI* susceptibility weighted imaging, *MT* magnetization transfer, *PD* proton density, *N.s*. not stated, *PD* (patient type) Parkinson’s disease, *MS* multiple sclerosis

The thalamus (Th), a structure that contains roughly four times less iron than the SN (Hallgren and Sourander 1958) is visualized with a much wider range of MRI sequences (Fig. 8d). A T_2_* based contrast is used most frequently which is surprising given the lower iron concentrations in the Th, but is closely followed by T_1_ based sequences.

#### Optimal MRI Contrast

There are a number of studies that explicitly state that one MRI contrast is superior to other sequences for the identification or parcellation of the SN, STN, or Th. There were 7 papers for the SN (Abduljalil et al. 2003; Abosch et al. 2010; Deistung et al. 2013a, b; Eapen et al. 2011; Schäfer et al. 2012; Shmueli et al. 2009; Khabipova et al. 2015; Kerl et al. 2012), 6 papers for the STN (Abosch et al. 2010; Schäfer et al. 2012; Kerl et al. 2012; Deistung et al. 2013b; Zeineh et al. 2014; Alkemade et al. 2017), and 6 papers that compared sequences for the Th (Abduljalil et al. 2003; Hammond et al. 2008a; Abosch et al. 2010; Deistung et al. 2013b; Tourdias et al. 2014; Kanowski et al. 2014). For the SN, the consensus for visualization seems to be that either a T_2_* or SWI based sequence is optimal, which are highly similar contrasts. For the STN, this is not as clear as there are roughly an equal number of studies that prefer T_2_*, SWI or T_2_ based images. The Th was preferentially visualized using a T_2_* contrast (see Table 3).

**Table 3:** Preferred MRI sequence for the visualization of the SN, STN, and Th

Structure	T_1_	T_2_	T_2_*	SWI	Other
SN	–	–	6	4	–
STN	–	–	2	4	1
Th	2	–	3	2	–

## Discussion

The subcortex can be parcellated into a large number of anatomically distinct structures (Federative Committee on Anatomical Terminology 1998). Only approximately 7% of these known structures are incorporated in standard anatomical MRI atlases (Alkemade et al. 2013). However, by reviewing the literature that utilized UHF MRI to visualize the subcortex, it became apparent that the number of observed subcortical structures is considerably larger. Specifically, at least 163 unique subcortical structures are identifiable in individual space using UHF MRI. We have provided R code to enable the reader to explore the use of UHF MRI for a given structure. A reader interested in structure ‘A’ can now obtain a list of the papers identifying this structure and the resolutions and methods used to do so.

The ability of UHF MRI to identify a large number of subcortical nuclei in individual space is of the utmost importance given the anatomical variability that exists across individuals (Mazziotta et al. 1995; Amunts et al. 1999; Uylings et al. 2005; Daniluk et al. 2009; Keuken et al. 2014). This anatomical variability is far from static as a number of factors including gene–environment interactions, healthy aging, and disease all influence individual anatomy over time (Thompson et al. 2001; Raz 2005; Lenroot and Giedd 2008; Daniluk et al. 2009; Keuken et al. 2013, 2017). These factors question the validity of using anatomical atlases which fail to incorporate anatomical variability or are not specific for an age group or clinical population (Devlin and Poldrack 2007; Alho et al. 2011).

### The Clinical Use of UHF

There are numerous recent reviews highlighting the substantial benefits of UHF MRI in a clinical setting (Cho et al. 2010b; Beisteiner et al. 2011; Duchin et al. 2012; Plantinga et al. 2014; Kraff et al. 2014; Trattnig et al. 2015, 2016; Gizewski et al. 2015). A number of studies have directly compared clinically utilized 1.5 and 3.0T field strengths to UHF MRI, showing UHF MRI results in an improved visualization across a number of patient groups and structures (Peters et al. 2007; Cho et al. 2008a, 2010b, 2010a, 2011a; Hammond et al. 2008a; Kollia et al. 2009; Yao et al. 2009; Zwanenburg et al. 2009; Abosch et al. 2010; Blazejewska et al. 2013; Chalifoux et al. 2013; Derix et al. 2014; Saranathan et al. 2014; Cosottini et al. 2015). Based on our own review, it is clear that UHF MRI is already frequently used to visualize subcortical structures in a clinical setting for populations such as Parkinson’s Disease, Alzheimer’s Disease, and Multiple Sclerosis. The benefit of UHF MRI in a clinical setting can be illustrated by its use with regards to preoperative planning for Deep Brain Stimulation (DBS) procedures as a treatment for PD patients. DBS is a surgical procedure where an electrode is inserted into the STN with the goal of reducing the motor symptoms of the disease, while simultaneously minimizing the occurrence cognitive and limbic side-effects known to affect a number of patients (Limousin et al. 1995; Temel et al. 2005). The development of these side-effects can partially be attributed to the suboptimal placement of the electrode in the STN (Kleiner-Fisman et al. 2006; Cakmakli et al. 2009; Paek et al. 2011). Given that the location of the STN changes with both age and disease (Dunnen and Staal 2005; Kitajima et al. 2008; Keuken et al. 2013, 2017; Mavridis et al. 2014; Pereira et al. 2016) it is crucial to visualize such a structure as accurately as possible per individual, which is why the superior visualization of UHF MRI is so valuable to DBS. The same logic can be passed to alternative neurosurgical interventions such as tumor delineation and removal, proton beam, gamma knife and radiation therapies which all require precise anatomical visualization, best afforded by UHF MRI (Forstmann et al. 2017b).

#### Optimal MRI Sequence per Structure

Optimal MRI sequences providing sufficient Contrast-to-Noise Ratio (CNR) are essential for clinical research. It is crucial to visualize the structure of interest while maintaining a clinically feasible scanning time. Therefore, given that different tissues require different MR sequences and parameters, it is important to experimentally determine the optimal sequence for each structure of interest (Marques and Norris 2017).

To highlight the variability of preferred sequences, the studies that used multiple MRI sequences to visualize the SN, STN, and Th were compared. Based on the literature review, the preferred contrast to visualize any of these three structures, even the Th is a T_2_* sequence (Abduljalil et al. 2003; Hammond et al. 2008a; Shmueli et al. 2009; Abosch et al. 2010; Eapen et al. 2011; Schäfer et al. 2012; Kerl et al. 2012; Kerl 2013; Deistung et al. 2013a, b; Gizewski et al. 2013; Tourdias et al. 2014; Zeineh et al. 2014; Saranathan et al. 2014; Kanowski et al. 2014; Khabipova et al. 2015). Such T_2_* sequences have been used in PD patients to investigate pathological alterations occurring in the SN dopaminergic system [e.g., (Cho et al. 2010b, 2011b; Kwon et al. 2012)]. Particularly at high UHF MRI the use of a T_2_* weighted sequence for a volumetric study is however not trivial. Pronounced B_0_ inhomogeneities lead to additional dephasing which may result in signal dropouts especially in regions with high iron content. Additionally, a major difficulty in interpreting T_2_*-weighted gradient-echo data is that the dependence of the signal on the tissue susceptibility is a non-local effect, i.e., the signal within a voxel is not only from effected by sources within but also from neighboring sources outside that voxel. Therefore, T_2_* hypointensity and phase contrast in gradient-echo techniques are not directly reflective of local tissue properties (Schäfer et al. 2009) which can effect volumetric measurements (Chandran et al. 2015). Shorter TE acquisition are preferable for volumetric measurements in terms of edge fidelity, but do not have the high contrast associated with midrange TE’s. What the optimal sequence is for the other subcortical structures is unclear from the current available publications and will probably differ from the SN, STN, and Th due to differences in tissue properties, most notably the lower concentrations of iron.

It should also be noted that these comparison studies should be viewed with the ongoing development of MRI contrasts such as QSM in mind (Marques and Norris 2017). QSM is a novel post-acquisition processing technique where the susceptibility of the tissue is quantified by estimating the magnetic field distribution and solves the inverse problem from field perturbation to magnetic susceptibility, while removing the background field contribution (Schweser et al. 2011, 2016). As such the QSM suffers less from non-local effects as described above which makes it an interesting contrast for volumetric studies of iron rich nuclei [e.g. (Liu et al. 2013; Alkemade et al. 2017)].

#### Quantitative Maps

Most of the included UHF MRI studies use standard MRI sequences that are (mainly) weighted for a certain contrast mechanism as opposed to a quantitative map, of, e.g., T_1_ or T_2_* relaxation. This is unfortunate as there are several clear advantages to quantitative MRI (qMRI) over standard weighted sequences (Weiskopf et al. 2015). One of the benefits of qMRI is that the quantitative maps can be used to generate bias-free weighted images [e.g. (Renvall et al. 2016)]. Another benefit of quantitative maps is the possibility of assigning a physical meaning to the intensity value of the image and therefore being able to provide biologically and spatially specific information (Weiskopf et al. 2015; Ropele and Langkammer 2016). For instance, T_1_, the parameter describing the spin–lattice relaxation, has been used as a proxy for myelin content (Koenig 1991; Stüber et al. 2014; Lutti et al. 2014; Dinse et al. 2015), whereas T_2_*, the parameter describing the spin–spin relaxation in combination with field inhomogeneity, and especially QSM are thought to be informative for iron concentration (Fukunaga et al. 2010; Lee et al. 2010; Cohen-Adad et al. 2012; Stüber et al. 2014).

One of the downsides of qMRI is that the acquisition time of a quantitative map is usually longer than standard weighted MRI. However, this can be solved by combining different contrast mechanisms into one data acquisition enabling quantification of multiple MRI parameters within a clinically acceptable time (Weiskopf et al. 2013). The advantage of having multiple contrasts is that each contrast contains complimentary anatomical information that can be used to inform segmentation algorithms, such as the multimodal image segmentation tool [MIST, (Visser et al. 2016a, b)].

### Reporting the Demographic and MRI Protocol Values

A critical note needs to be made regarding the lack of details reported in the included papers. A substantial number of studies fail to report basic demographic information of the measured subjects. At times information regarding the exact age, gender ratio, and whether the participant is healthy is missing. This is problematic as age and disease can have substantial effects on the biological properties of the brain (Minati et al. 2007; Aquino et al. 2009; Fritzsch et al. 2014; Lorio et al. 2014; Visser et al. 2016b). In other cases, essential information regarding the MRI protocol such as field of view, matrix size, or voxel size is missing or incomplete. This hinders the reproducibility of these studies and makes it challenging to implement their sequences and protocols. As such it should be recommended that groups adhere to the guidelines on reporting neuroimaging studies (Poldrack et al. 2008; Nichols et al. 2016).

### Challenges of UHF MRI

An obvious limitation of UHF MRI is the limited accessibility. Of the approximately 36,000 MRI scanners available worldwide, only ± 0.2% are UHF MRI scanners (Rinck 2016). Given the advantages for visualizing clinically relevant subcortical nuclei, this calls for an increase of UHF MRI scanner sites but we acknowledge the substantial higher purchasing and running costs of a UHF MRI scanner. A more technical challenge with UHF MRI are the B_0_ and B_1_ field inhomogeneities which increase with field strength resulting in local signal intensity variations and signal dropout (Truong et al. 2006a; van der Zwaag et al. 2015). While B_0_ and B_1_ field inhomogeneity remains an active field of research, substantial progress has already been made in overcoming these problems (van der Zwaag et al. 2015; Yarach et al. 2016; Sclocco et al. 2017). For the subcortex, the absence of nearby air–water interfaces for most of the subcortical structures means that B_0_ inhomogeneities are a relatively minor problem. B_1_ inhomogeneities are more problematic. While the standard single-channel transmit/32-channel receive coils have a relatively favorable transmit B_1_ pattern with highest achieved flip angles in the middle of the brain, the receive profile of the array coils means that SNR is rather lower in the midbrain than in the cortex.

While the spatial resolutions achieved by in vivo UHF-MRI are impressive, on its own, it is not able to deliver the anatomical resolution needed to visualize all structures known to be present in the human brain. At present, the combination of neuroimaging and post mortem staining’s are still needed to create a complete and comprehensive picture of the human brain in its entirety (Yang et al. 2013; Amunts et al. 2013; Forstmann et al. 2017a). An example of such a combination has been given by Ding and colleagues (Ding et al. 2016). Here they used a single post mortem brain, which was structurally scanned with 7.0T and subsequently further processed using various staining techniques. A staggering 862 cortical and subcortical areas were manually segmented and aligned to the structural MRI scans. Given that it is not yet possible to fully automatize such a pipeline nor translate it directly to the individual in vivo brain, these efforts will not quickly result in a tool to identify the structures per individual brain. However, what such a multi-modal atlas could do is to provide shape, intensity, and spatial relationship priors for automatic segmentation methods (Bogovic et al. 2013; Kim et al. 2014; Visser et al. 2016a, b).

A final limitation of UHF MRI utility is that until recently the standard FDA approval for clinical scanning only went up to 3.0T (van Osch and Webb 2014). This restriction does not seem to be based on safety concerns, as the risks associated with UHF MRI up to 8.0T are similar to 1.5 and 3.0T (Administration 2003; van Osch and Webb 2014). This limitation has hindered the use of UHF MRI in standard clinical practice which, given the clear clinical advantages, is unfortunate (Kraff et al. 2014; Trattnig et al. 2015). This limitation has been recently been resolved as the newest generation of 7.0T systems (e.g., the Siemens 7.0T MAGNETOM Terra system) has both CE and FDA clinical approval (Heimbach 2015; Healthineers 2017a, b). This might result in more institutes having a larger interest in investing in UHF MRI scanners, increasing the accessibility for clinical and non-clinical research.

### Future Development

**Fig. 9:**
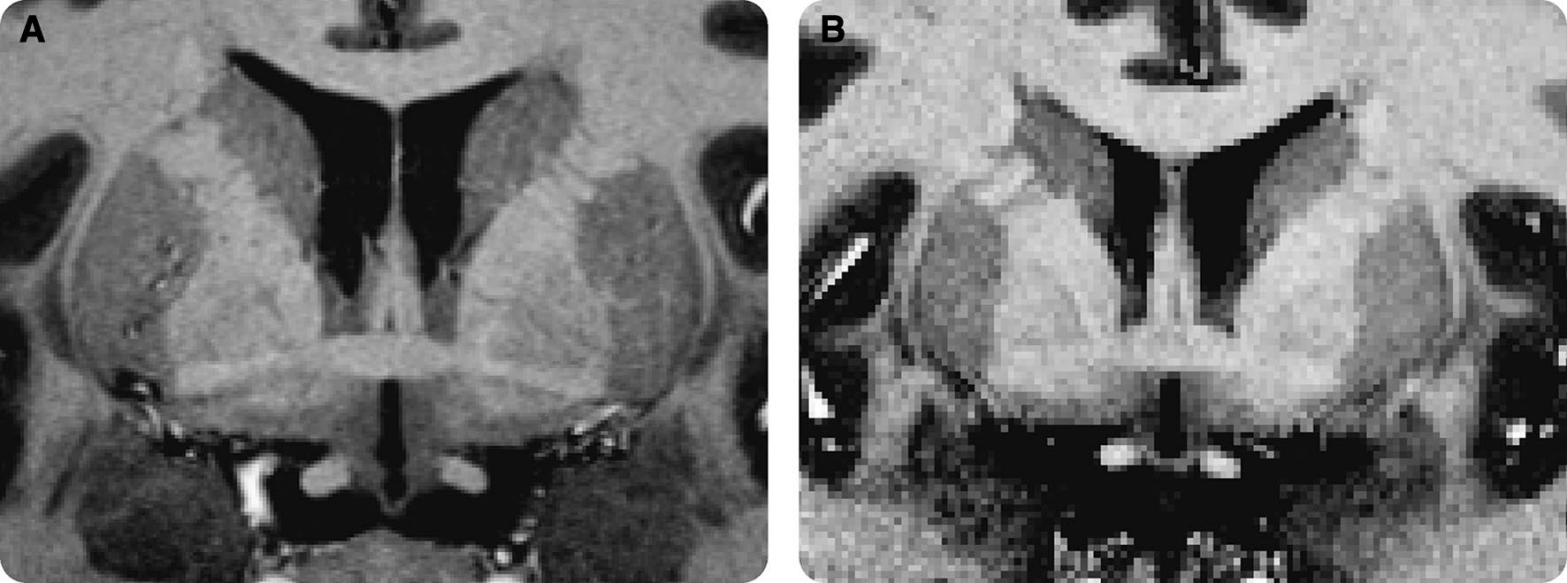
Structural MP2RAGE whole brain volumes with or without retrospective motion correction. **a** A MP2RAGE whole brain volume that was acquired with 0.35 mm isotropic voxel resolution using Fat-Navs for retrospective motion correction. The image is based on an average of 4 scans which were registered using trilinear interpolation. The MRI data is made freely available and described in Federau and Gallichan (2016). **b** A single MP2RAGE whole brain volume that was acquired with 0.7 mm isotropic voxel resolution, with no motion correction. The MRI data is made freely available and described in Forstmann et al. (2014). The MP2RAGE in panel A has a voxel volume that is 8 times smaller than in panel B. This difference in voxel size results in a substantially lower PVE

As the voxel sizes continue to decrease, involuntary subject motion becomes an increasing challenge, to the extent that muscle relaxation, cardiac pulsation, respiratory motion and swallowing have a measurable effect on the image quality (Herbst et al. 2013; Stucht et al. 2015). A possible solution for this would be prospective motion correction (PMC), where the MR gradient system is adjusted in real time to ensure that the brain remains in the same location in the imaged volume (Maclaren et al. 2012). PMC has been used in combination with UHF MRI and results of whole brain MP2RAGE scans with an isotropic resolution of 0.44 mm have been presented (Stucht et al. 2015). One of the downsides of PMC is that for the currently commercially available systems additional hardware is necessary to track the motion of the brain (Maclaren et al. 2012). Another possibility would be to use MR-based motion measures such as fat image navigators (fat-navs) (Gallichan et al. 2015; Federau and Gallichan 2016). Fat-navs are interleaved acquired high contrast images of the sub-cutaneous fat and bone marrow of the skull and can be used to estimate and correct head motion. Using these fat-navs, whole brain MP2RAGE scans with an isotropic resolution of 0.35 mm have been acquired at 7T (Stucht et al. 2015). The advantage of such high spatial resolution is that certain anatomical details such as the grey matter islands between the putamen and caudate become much more visible [see Fig. 9 for a visual comparison between two whole brain MP2RAGE datasets of which one used fat-Navs and higher spatial resolution. Data is provided by (Forstmann et al. 2014; Stucht et al. 2015; Federau and Gallichan 2016)].

## Conclusion

The number of UHF MRI sites are steadily increasing as there are several advantages over lower field MRI such as intrinsic higher SNR and increased CNR. With the increase of field strength, it becomes possible to visualize small subcortical structures and their subnuclei which are challenging to localize. This is illustrated in this review by the fact that UHF MRI, with a wide range of imaging approaches, has been able to identify 169 subcortical structures in the individual brain. Some of these concern subdivisions in structures that were only identifiable as a whole at lower fields. It should however be noted that most of these structures were only identified in a single publication. This is substantial progress, but also emphasizes the amount of work yet to be done to find a comprehensive imaging approach to parcellate the subcortex per individual. With the large efforts currently directed at UHF sequence development (Marques and Norris 2017) it seems especially likely that the number of identifiable structures will increase further.
